# Development of microsatellite markers and detection of genetic variation between *Goniozus* wasp populations

**DOI:** 10.1093/jis/14.1.43

**Published:** 2014-01-01

**Authors:** Sahand K. Khidr, Ian C.W. Hardy, Tania Zaviezo, Sean Mayes

**Affiliations:** 1 School of Biosciences, University of Nottingham, Sutton Bonington Campus, Loughborough, LE12, 5RD, UK; 2 Departamento de Fruticultura y Enología, Facultad de Agronomía e Ingeniería Forestal, Pontificia Universidad Católica de Chile, Casilla 306 – 22, Santiago, Chile

## Abstract

Molecular genetic markers reveal differences between genotypes according to the presence of alleles (the same or different) at target loci. Microsatellite markers are especially useful co-dominant markers that have been used in a wide range of studies to elucidate the population structure and dynamics of a range of organisms, including agriculturally beneficial insects such as parasitic wasps (parasitoids). In the present study, twelve primer pairs were designed for the south Asian , *Goniozus nephantidis* (Muesebeck) (Hymenoptera: Bethylidae), and 24 for its New World congener, *Goniozus legneri* Gordh, parasitoids of the larvae of the lepidopteran coconut pest *Opisina arenosella* Walker (Lepidoptera: Crytophasidae) and other lepidopteran pests, respectively, in order to investigate polymorphism within and between populations. The wasps fingerprinted were a total of 85 *G. nephantidis* and *G. legneri* , including individuals belonging to three putatively different strains of *G. legneri* . Annealing gradient tests (50-65°C) were conducted to study the quality of the PCR amplification across an annealing temperature gradient using a mixed genotype DNA template from each species separately. Seven primer pairs, which amplified clear products of approximately the expected size of *G. nephantidis* and 18 of *G. legneri* , were then selected for capillary analysis for fragment size determination on a *Beckmann* CEQ 8000. Neither *G. nephantidis* nor *G. legneri* were polymorphic within populations. However, there were six primer pairs that did show polymorphism between *G. legneri* populations that originated from different geographical areas within South America (Uruguay and Chile). Furthermore, one primer pair revealed diversity between the two strains collected within Chile. One of the markers was subsequently used to provide unbiased assessment of primary sex ratio in *G. legneri* .

## Introduction

Among the natural enemies of agricultural insect pests, hymenopteran parasitoids are one of the most important classes of biological control agents and are also widely used in studies of evolutionary ecology and basic population biology ( Godfray 1994 ; Jervis 2005 ; Hochberg and Ives 2000 ; Wajnberg et al. 2008 ; Hardy et al. 2013 ). The distributions and population structures of parasitoids are influenced by a wide range of factors, such as geological and geographical components, ecological processes, and evolutionary and genetic aspects ( Zink 2002 ; Bond and Stockman 2008 ). Successful population genetic, ecological, and evolutionary studies can be achieved through the availability of suitable molecular markers, which are important indicators of relationships between both individuals and populations ( Carvalho 1998 ). Such markers can reveal differences between genotypes through the application of a range of random markers not linked *a priori* to traits.

Among the classes of widely used genetic markers are ‘microsatellite’ markers, also known as simple sequence repeats (SSRs). These consist of short, repeated units of around two to six base pairs in length with an array that can be up to 200 bp long and are found in both coding and non-coding regions in all prokaryotic and eukaryotic genomes (Tautz 1989; Arcot et al. 1995 ; Beukeboom and Zwaan 2005 ). To date, they have been developed in a number of parasitoids, especially braconids ( Baker et al. 2003 ; Anton et al. 2006 ; Lozier et al. 2006 ).

Microsatellite markers, which are co-dominant ( Loxdale and Lushai 1998 ), have many advantages over other marker types because not only do they generally have a high number of alleles per locus, which can identify polymorphism, but they can detect high levels of heterozygosity and have high mutation rates ( Hancock 1999 ). As such, microsatellite markers have been used to determine the genetic diversity and differentiation between populations through measuring the degree of heterozygosity in parasitoid species (e.g. 0.04–0.44 in *Cotesia melitaearum* , 0.171–0.629 in *Neotypus melanocephalus* , and 0.170–0.367 in *Lysiphlebus hirticornis* ; Kankare et al. 2005 ; Anton et al. 2007 ; Nyabuga et al. 2010 ) as well as the degree of gene flow and dispersal between populations ( Avise 1994 ; McCoy et al. 2001 ; Molbo et al. 2003 ; Kankare et al. 2005 ; Zavodna et al. 2005; Drescher et al. 2010 ; Nyabuga et al. 2010 ).

In the present study, we designed a suite of microsatellites for screening two species of bethylid wasps for genetic polymorphisms within and between populations. In principle such markers could also prove useful for pest control applications ( Aebi et al. 2008 ; Ugelvig et al. 2008 ; Lozier et al. 2009 ; Zygouridis et al. 2009 ; Nicholls et al. 2010 ; Lavandero et al. 2011 ), evaluating the effect of kinship on social behaviors ( Lizé et al. 2012 ), and for measuring population parameters, such as levels of inbreeding, which have not been directly evaluated but are important in the understanding of reproductive decisions ( Hardy and Cook 1995 ; Hardy et al. 1998 , 1999, 2000). The first direct application of these markers has been to provide assessment, as described elsewhere ( Khidr et al. 2013 ), of the sex of individual parasitoid eggs in order to evaluate maternal sex allocation without the biasing influence of developmental mortality.

The Bethylidae is a family of parasitoid hymenopteran wasps that has been thought to comprise four extant subfamilies, Bethylinae, Epyrinae, Pristocerinae, and Mestitiinae (Evans 1964), with over 2000 described species ( Gordh and Móczár 1990 ). Recently, the higher level phylogeny of bethylids has been estimated using molecular data from 33 species, resulting in a split of the subfamily Mestitiinae into two separate subfamilies, the Mestitiinae and the Cephalonomiini ( Carr et al. 2010 ). Bethylid wasps attack almost exclusively the immature stages of coleopterans and lepidopterans, many of which are pests of important agricultural commodities such as coffee, coconut, sugarcane, apple, walnut, and almonds ( Gordh 1982 ; Batchelor at al. 2005 ; Venkatesan et al. 2007 ; Zaviezo et al. 2007 ). In this study, we have focused on *Goniozus nephantidis* (Muesebeck) (Hymenoptera: Bethylidae), a parasitoid of the lepidopteran larvae of the coconut pest *Opisina arenosella* Walker (Lepidoptera: Crytophasidae) in the Indian subcontinent, and *G. legneri* Gordh, a parasitoid of several New World lepidopteran pests of walnuts, pistachio nuts, almonds, and apples ( Steffan et al. 2001 ; Garrido et al. 2005 ; Zaviezo et al. 2007 ).

Both G *. nephantidis* and *G. legneri* have each been used in biocontrol programs (Dharmaraju 1963; Legner and Silveira-Guido 1983 ; Gothilf and Mazor 1987 ; Lyla et al. 2006 ) and in a range of behavioral ecological studies (e.g., Hardy and Cook 1995 ; Goubault et al. 2006, 2007 ; Humphries et al. 2006 ; Bentley et al. 2009 ; Lizé et al. 2012 ). Their basic life histories are similar; both are gregarious idiobiont ectoparasitoids exhibiting sub-social behavior, such as maternal care and defense of the developing brood ( Hardy and Blackburn 1991 ; Bentley et al. 2009 ), and appear to conform closely, but probably not exactly, to single foundress local mate competition ( Hamilton 1967 ; Hardy and Cook 1995 ; Hardy et al. 1998, 1999, 2000). Males usually emerge before females and have sufficient capacity to inseminate their sisters ( Hardy et al. 1999 , 2000), and, as with many other bethylids, the sex ratios of these species are generally female biased with a low degree of sex ratio variation between broods (sub-binomial variance: Green et al. 1982 ; Hardy and Mayhew 1998 ; Hardy et al. 1998 ; Khidr et al. 2013 ).

## Materials and Methods

### Parasitoid origins and cultures

The culture of *G. nephantidis* used in this study had been maintained in the laboratory for more than 20 years on the facultative host *Corcyra cephalonica* Stainton (Lepidoptera: Pyralidae), as described in Lizé et al. (2012) . *Corcyra cephalonica* was also used as the facultative host for *G. legneri* . Three strains of *G. legneri* were used. One, termed strain ‘U’, was obtained from a commercial insectary in the USA and kept in our laboratory for more than eight years. The original material is believed to have been collected from southern Uruguay in 1978 ( Gordh 1982 ; Gordh et al. 1983 ; Legner and Silveira-Guido 1983 ). Two further strains of *G. legneri* were brought to our laboratory in May 2009 from Santiago, Chile. One strain was collected directly from walnut trees and was termed ‘C-field’, and the other strain was termed ‘C-lab’, as it had been maintained in a Chilean insectary for several years following collection from a field site near Santiago ( Zaviezo et al. 2007 ). All cultures were maintained in a constant environment room at 25–27°C, 12:12 L:D, and with high relative humidity maintained by an ambient temperature water bath.

### Design and preparation of the primers

Microsatellite-enriched genomic libraries were created essentially according to Kloda et al. (2004) , with the final sequencing step performed using barcoded adaptors and a 1/16th run of non-titanium reagents Roche 454 pyrosequencing (as part of a mixture of 9 different libraries) ( www.454.com ). The generated Fasta files were separated *in silico* to identify the individual libraries, and those for *G. legneri* and *G. nephantidis* were searched for microsatellite motifs using the MISA.pl script ( http://pgrk.ipk-gartersleben.de/misa/misa.html ). Primer pairs flanking the simple sequence repeats were designed either by Primer 3 ( Rozen and Skaletsky 2000 ) and/or WebSat ( Martins et al. 2009 ) for *G. legneri* ( Table 1 ) and for *G. nephantidis* ( Table 2 ). Primers were synthesized by Eurofins MWG Operon ( www.operon.com ) with a forward primer 5’ extension consisting of the M13 sequence to allow fluorescent labeling of the final product through a three primer reaction ( Schuelke 2000 ), and prepared to 1000×concentration using Sigma-Aldrich ( www.sigmaaldrich ) molecular biology grade water (to create primer stocks of 200 pmol/µL). After vortexing and spinning, tubes were placed on ice for 30 min. Primers were kept in a freezer at -20°C, and to produce a 10×primer stock, 5µL of the 1000×stock was mixed with 495 µL sterile distilled water for both forward and reverse primers in separate tubes on ice. The third primer (M13;TGTAAAACGACGGCCAGT-3’) was ordered from Sigma-Aldrich and labeled with dye D4 (blue; WellRed dyes).

**Table 1.**
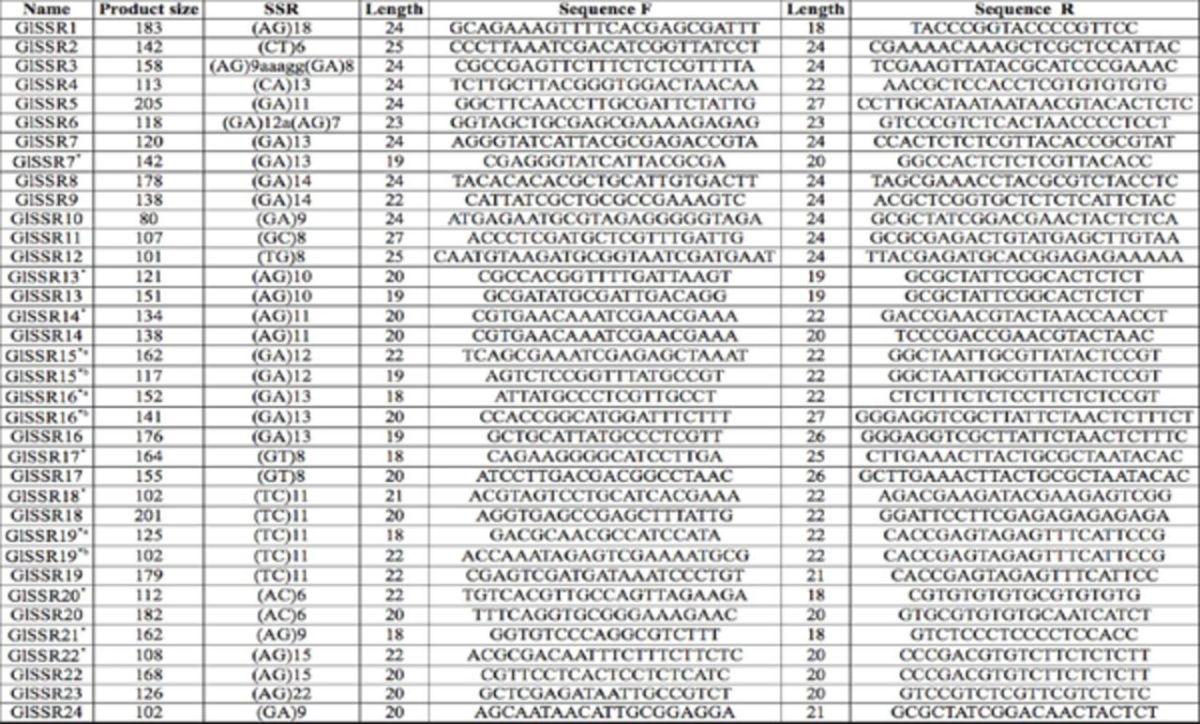
Primers designed for *Goniozus legneri.*

*Denotes primers designed using WebSat. In some cases there were two versions designed for a particular fragment length, denoted by a or b. The remainder of the primers were designed using Primer 3.

Note: The expected product sizes do not include the M13 extension primer, which adds 18 bases to the product size.

**Table 2.**
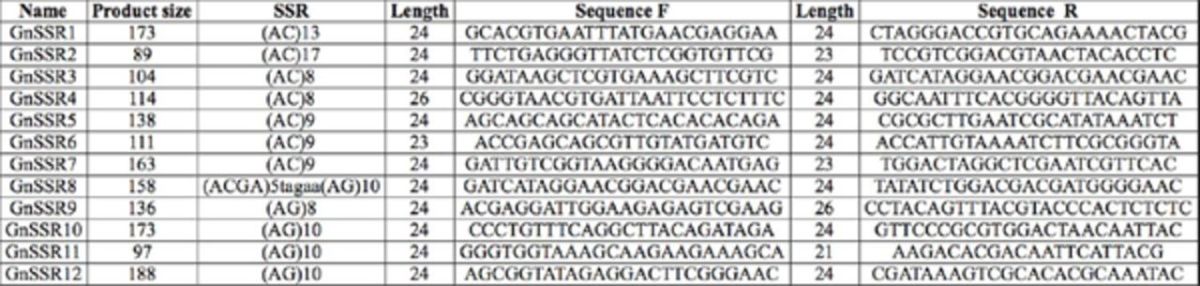
Primers designed for *Goniozus nephantidis.*

Note: the expected product sizes do not include the M13 extension primer, which adds 18 bases to the product size.

### DNA extraction

A sample size of 85 adult individuals was used for capillary testing. Seventeen individuals of *G. nephantidis* were examined, plus a total of 68 individuals for the different strains of *G. legneri* (25 of ‘U’ strain, 22 of ‘C-lab’ strain, and 21 individuals of the ‘C-field’ strain). In addition, five pooled samples of 20 individuals were used for the annealing gradient test. Individual adult females or pooled adult samples were placed in 1.5 mL Eppendorf tubes (Sarstedt, www.sarstedt.com ) and then immersed into liquid nitrogen and crushed using a mini pestle to start the extraction. Genomic DNA was then extracted either by using a GenElute plant Genomic kit (Sig-ma-Aldrich) or by following, with some modifications, methods given in Sambrook et al. (1989) and Vogler and Desalle (1993) before elution/resuspension into 50-60 µL of sterile distilled water and storage at -20 ^o^ C.

### Polymerase chain reaction (PCR)

PCR reactions were performed in either a Thermo Hybaid Express PCR machine ( www.thermohybaid.com ) or in an ABI PCR 9700 Thermocycler machine (Applied Biosystems, Life Technologies, www.lifetechnologies.com ). The Thermo Hybaid Express PCR was used for annealing gradient tests and run with a 15°C gradient by using a total volume of 20 µL for one reaction through mixing different components consisting of 2 µL of 10×forward primer and 2 µL of 10×reverse primer (2 pmol/µL final); 2 µL of 10×PCR buffer; 0.16 µL of dNTP’s (mixed dNTP 25 mM final concentration per nucleotide); 2 µL of DNA template (mixture of many individuals); 0.10 µL of *Taq* DNA polymerase (5 units/µL), and 11.7 µL of sterile distilled water. Master mixes were prepared, where possible, to decrease the effects of pipetting error.

Thus, the optimal annealing temperatures for each of the primer pairs used were determined according to following program: an initial 3 minutes denaturation at 94°C, followed by 35 cycles of 1 minute at 94°C (denaturation), 1 minute at 50–65°C (annealing), and 72°C for 2 minutes (extension), with a final extension of 72°C for 10 minutes at the end of the program.

For genotyping of individual samples amplified in the ABI PCR 9700 Thermocycler, the aforementioned program was used, but the determined optimum temperature for the annealing step was 60°C for the majority of the primers unless otherwise stated. PCR reactions consisted of 0.2 µL of 10×forward primer and 2 µL of 10×reverse primer (2 pmol/µL final), 2 µL (10×) PCR buffer, 0.16 µL dNTP’s (each in a 25 mM final concentration), ~ 0.05 µL M13 Blue *Taq* of 1000×(53.8 nM concentration), 2 µL of individual genomic DNA (~ 5 ng/µL), 0.10 *Taq* DNA polymerase (5 units/µL), and 13.5 µL sterile distilled water. Thus, a fluorescently-labeled M13 tail sequence was added to the 5’-end of the forward primer ( Schuelke 2000 ) to be used for capillary sequencing.

### Agarose gel electrophoresis

Samples to be loaded onto the gel were mixed with 6×gel loading blue buffer (Promega, www.promega.com ) in the ratio of one part sample to one part loading buffer. Loading buffer was added to each well of the plates from the PCR machine and was spun briefly. Thereafter 10 µL from each well was loaded onto a submerged gel that consisted of 2% agarose (molecular grade, Bioline, www.bioline.com ) prepared in 0.5×TBE (Tris-Borate-EDTA, pH 8.0) buffer, followed by addition of 2 µL of ethidium bromide stock before pouring (10 mg/mL stock; Promega). Each primer pair reaction was loaded onto one row of the gel (each primer pair having 12 reactions across a 15°C annealing gradient). Alongside, an appropriate size marker (5 µL of 2-log DNA ladder; New England Biolabs, www.neb.com ) was loaded in the first lane of each primer pair, and the gel was run at 90 V for approximately 1 hour. Following electrophoresis, bands were visualized and photographed under UV-light in a Bio-Rad Gel Doc 2000 gel box ( www.bio-rad.com ). Quantification test of DNA templates and *Goniozus* individuals’ DNA extractions were carried out by comparison with known uncut lambda DNA (BioLabs) (50 ng/µL) loaded in the amounts of 10 µL, 5 µL, 2.5 µL, and 1.5 µL to provide a fluorescence comparison with the unknown samples. The 1% agarose gel was run at 90 V for 75 min, then different individuals of both species were quantified and tested for DNA integrity by ensuring that genomic samples largely ran at limiting mobility. Good quality samples were used as DNA templates.

### Capillary sequencing: Preparing fragment samples for analysis

The CEQ 8000 Fragment Analysis Software Version 8 (Beckman Coulter, www.beckmancoulter.com ) was used to measure and analyze the PCR product fragment sizes. For the preparation of the sample in half-reactions, for each row of eight samples, 215 µL of SLS (sample loading solution) was added to 2 µL of SS 400 (standard size) mixed by vortexing and spun briefly. 27 µL of this mixture was then added to each well in the row, and 2 µL of multiplexed PCR product was later added. The mixture in each well was overlaid immediately with a drop of mineral oil and placed in the CEQ machine. Later, cluster analysis between different populations of *G. legneri* was performed using the Multi-Variate Statistical Package version 3.2 (MVSP; Kovach Computing Services, www.kovcomp.co.uk ).

## Results

### Primer design in the Microsatellite library

A genomic library consisting of 273 sequences containing microsatellite motifs was screened to design primers for *G. legneri* . Twenty-four of these sequences were considered to be clearly unique sequences with adequate flanking sequence length to design primer pairs flanking the repeat unit. The di-nucleotide (GA)n repeat was the predominant marker, followed by (AG)n and (TC)n, while the tri-and tetra-nucleotide microsatellites were frequently present as compound microsatellites. In *G. nephantidis* , there were 3356 microsatellites, of which 12 were chosen to design primers for the investigation of polymorphism within the population.

### Annealing gradient tests

**Figure 1.**
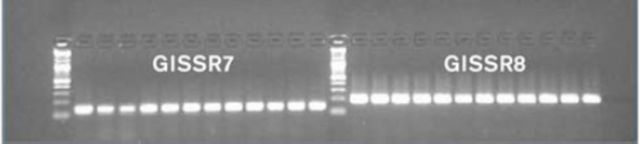
Annealing gradient for primers 7 and 8 of *Goniozus legneri* (primer labels correspond to those in Table 1 ). High quality figures are available online.

PCR analysis was performed to optimize annealing gradients for the 12 new *G. nephantidis* primer pairs and 24 primer pairs for *G. legneri* strains. Occasionally a number of primer pairs were designed to the same microsatellite repeat sequence to increase the probability of successful amplification. The process was repeated several times to test the reliability of the new primers. Representative results of the annealing electrophoresis gels for *G. legneri* primers are shown in Figure 1 , with the left hand column showing the 2-log DNA ladder (New England Biolabs).

Primers displaying clear bands in annealing tests were selected for PCR amplification and polymorphism testing. In addition, the best annealing temperature for each primer was recorded in order for it to be used for the PCR. According to the results of the annealing tests, the best temperature for all *G. nephantidis* primers was 60°C, except for primer GnSSR11 at 56°C. No amplification was observed for primers GnSSR1, 2, 3, 4, and 10 in the test. In *G. legneri,* the optimum temperatures were 54°C, 56°C, and 58°C for primers GlSSR 14, 5, and 22, respectively. The remainder of the primer pairs had optimal annealing temperatures close to 60°C, allowing simultaneous amplification in the same thermoblock. The following primers were excluded from further work: GlSSR1, 4, 6, 10, 19*b, 20*, 21*, 22*, and 23.

### DNA quality test

DNA quality tests were conducted for *Goniozus* that were to be used as a template, and samples were diluted for annealing tests. However, DNA preparations from individual wasps for use in PCR did not usually need dilution because concentrations were generally at or below 10 ng/µL.

### Second round of PCR runs

**Figure 2.**
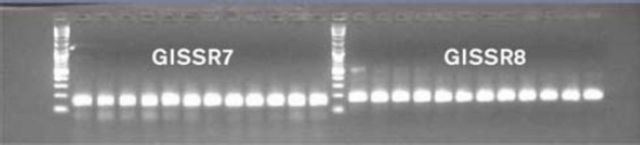
Gel plage of PCR products for primers 7 and 8 of *Goniozus legneri.* High quality figures are available online.

Primers chosen in the annealing test were amplified on the ABI PCR machine at different temperatures according to their annealing test optima. The gel electrophoresis results were visualized using UV light. Some primers were rejected before capillary testing due to lack of amplification in an annealing test or, if they amplified, not displaying clear/discrete single bands on the gel. PCR products of primers 7 and 8 amplified from *G. legneri* are shown in Figure 2 .

### Capillary sequencing

**Figure 3.**
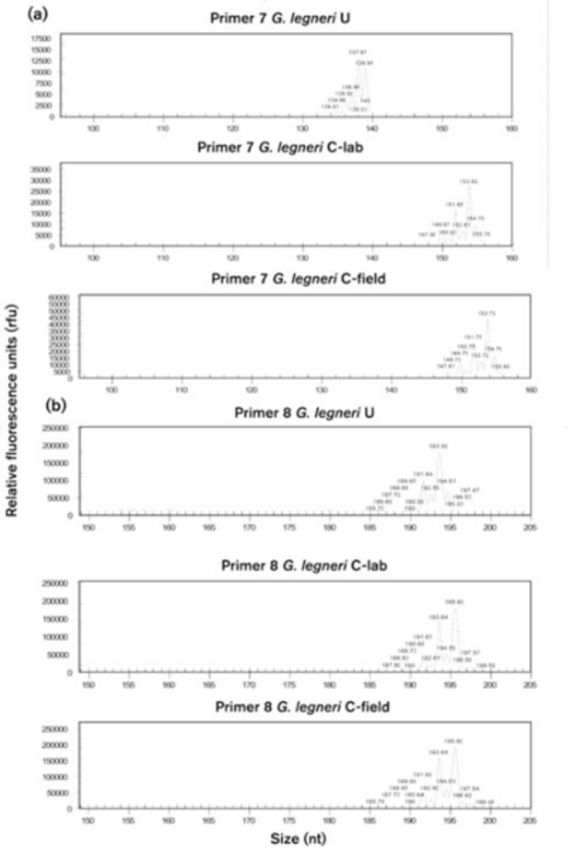
Examples of capillary tube analysis of primer 7 (A) and primer 8 (B) for *Goniozus legneri* strains. High quality figures are available online.

**Figure 4.**
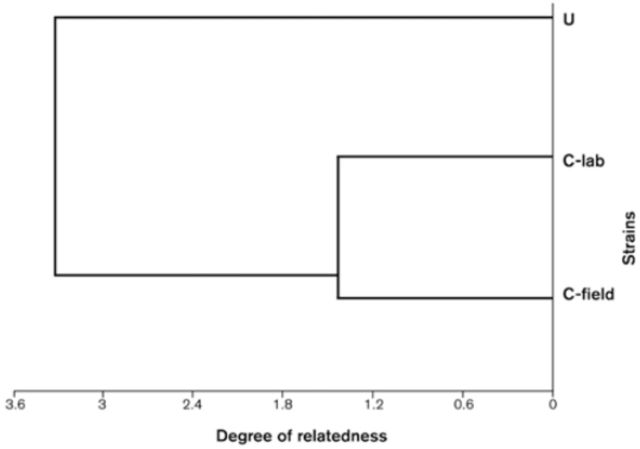
Dendogram showing relationships between and within strains of *Goniozus legneri.* High quality figures are available online

The results of the capillary fragment analysis were processed using the CEQ 8000 DNA sequencer to determine amplified product size. Neither *G. nephantidis* nor *G. legneri* were polymorphic within strains. Nonetheless, there were six primers that showed clear inter-strain polymorphism in *G. legneri* ( Table 3 ). For instance, primer GlSSR7 showed a large size difference between strains, while U-strain was 137 bp and both Chilean populations were 153 bp. Furthermore, primer GlSSR5 showed a different size allele at 228 bp for both U and C-lab strains, with 224 bp for the C-field strain ( Figure 3 ). Dendrogram analysis based on the overall microsatellite alleles patterns showed clear differences between populations from the two different geographical locations, Uruguay and Chile, as well as differentiation within the two strains collected in Chile (C-lab and C-field). However, both strains were more closely related to each other than either was to the Uruguay strain ( Figure 4 ).

**Table 3.**
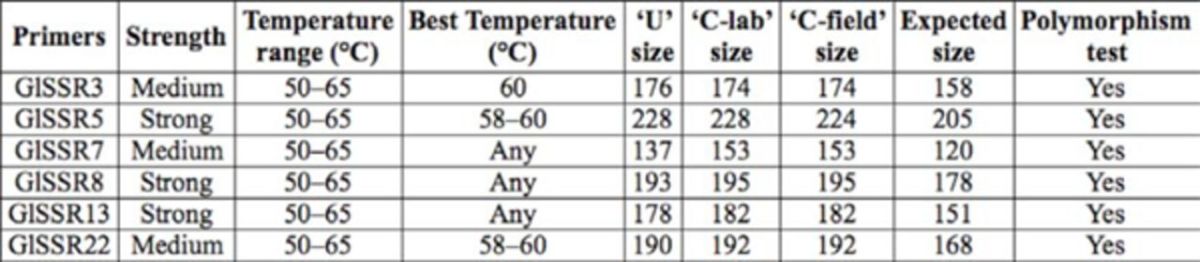
The six polymorphic primers for *Goniozus legneri* strains.

Note: the expected product sizes do not include the M13 extension primer, which adds 18 bases to the product size.

## Discussion

The main aim of this study was to develop molecular markers for bethylid wasps so that these markers could subsequently be utilized in evolutionary ecology as well as in applied agricultural research. In general, the percentage of amplified loci decreases with increasing genetic distance between the species tested, making such markers most suitable for closely-related species, such as congeners ( Hancock and Simon 2005 ; Barbara et al. 2007 ). Since there are well over 100 described species of *Goniozus* ( Gordh and Móczár 1990 ), such markers have considerable potential in many diverse studies, both pure and applied, on this genus of parasitoids.

The molecular data from this study reveal a lack of variation within strains of both *G. nephantidis* and *G* . *legneri* . Potential reasons for this include a loss of genetic diversity during laboratory maintenance (e.g., due to relatively small populations in culture, occasional population crashes, and the exposure of deleterious recessive genes in haploid males) ( Unruh et al. 1984 ; Shields 1993 ; Cook 1993 ; Henter 2003 ). However, this explanation would most likely apply to *G. nephantidis* and the U-strain of *G. legneri* , as these have been maintained in culture for many more years than the strains of *G. legneri* from Chile (C-lab and C-field). The fact that polymorphisms were not found within the much more recently collected Chilean strains suggests that lack of variation is not a result of genetic drift due to small population and time-in-culture effects. A more probable explanation is that there is limited genetic variation within each of the populations from which field collections were made. Genetic homozygosity can result from inbreeding because close relatives mate more frequently than expected by chance given the overall size of the population ( Henter 2003 ; Elias et al. 2010 ; Mazzi et al. 2011 ), and both species of *Goniozus* are known to exhibit high levels of pre-dispersal sibling mating in the laboratory ( Hardy et al. 1999 , 2000) and have sex ratios that largely conform to theoretical expectations under such ‘local mate competition’ ( Gordh et al. 1983 ; Hardy and Cook 1995 ; Hardy et al. 1998 ; Khidr et al. 2013 ). It is further known that *G. nephantidis* does not exhibit inbreeding depression in terms of effects on developmental mortality or sex ratio control ( Cook 1993 ). Nonetheless, to date the post-dispersal mating behavior of these two species has not been directly evaluated, although the natural mating systems of these wasps are likely to have a large effect on the evolution of their sex ratios ( Hardy 1994 ; Hardy and Cook 1995 ; Hardy and Mayhew 1998 ; Hardy et al. 1998 ). The lack of within-strain genetic polymorphism that was observed provides a degree of evidence that sibling-mating is the predominant feature of the natural mating system of both *Goniozus* species.

For *G. legneri* strains collected within the same geographical region of Chile, C-lab and C-field, an allelic difference was observed with just one primer of the six amplified. This could relate to the fact that the field strain was collected from carob moth larvae ( *Ectomye-lois ceratoniae* Zeller (Lepidoptera: Pyralidae)) feeding on walnuts, while the laboratory strain was derived from a mixture of individuals collected from both carob moth on walnuts and codling moth larvae *(Cydia pomonella* L. (Lepidoptera: Tortricidae)) feeding on apples ( Zaviezo et al. 2007 ; T. Zaviezo personal observation, I. Hardy personal observation). Genetic diversity in several other parasitoid species has been found to be associated with host and host plant species (e.g., Kavallieratos et al. 2004 ; Stireman et al. 2006 ).

Six of the primer pairs tested showed clear microsatellite polymorphisms between *G. legneri* strains (U and C-field). Genetic differences between these strains could arise due to differences in the species of insect host or host plant they were collected from (see above) and also to geographical differences ( Menken 1981 ; Ruiz-Montoya et al. 2003 ; Stireman et al. 2006 ; Pannebakker et al. 2008 ; Phillips et al. 2008 ; Lozier et al. 2009 ; Lavandero et al. 2011 ). Various biological traits and genetic diversity might associate with populations from different geographic localities due to geographic isolation, influenced by different climatic effects and thus different selection pressures ( Diehl and Bush 1984 ; Hopper et al. 1993 ; Thompson 1994 ; Goodisman et al. 2001 ; Hufbauer et al. 2004 ).

Establishing that there is genetic polymorphism within parasitoid species opens up a number of possibilities for the use of these markers. For instance, molecular markers have been used to show that the host searching behavior of different *Agathis* sp. n. populations was not affected by geographical structure and they have the ability to disperse for long distances ( Althoff and Thompson 2001 ), and reciprocal crossing of two geographically and host-species distinct strains of *Aphelinus albipodus* showed reproductive compatibility and no reduction in fecundity ( Wu et al. 2004 ). Furthermore, genetic relatedness is a crucial factor in the evolution of social behaviors between individuals (Hamilton 1964a, b; Mateo 2004 ; Lizé et al. 2006 ; Gardner and West 2007 ), and relatedness between insects can be usefully assessed using microsatellite markers ( Buczkowski et al. 2004 ; Trindl et al. 2004 ; Jaquiery et al. 2005 ; Drescher et al. 2010 ), leading to key insights into behaviors such as kin-based altruism and aggression assays ( Giraud et al. 2002 ; Tsutsui et al. 2003 ; Drescher et al. 2010 ; El-Showk et al. 2010 ). In our own study system, the development of microsatellite markers provides useful support for empirical work on kin recognition mechanisms ( Lizé et al. 2012 ), as they confirm the assumption that females from different strains of *G. legneri* derive from populations with different genetic backgrounds, and thus are not as closely related as are females from within the same strain. In general, genetic recognition cues will usually associate with the level of polymorphism ( Ratnieks 1991 ; Buczkowski et al. 2004 ), and the degree of aggressive behavior between encountered individuals is attuned to the level of genetic diversity recognition loci ( Giraud et al. 2002 ; Drescher et al. 2010 ).

Microsatellite markers have also been used in studies of hymenopteran sex ratios to identify the sex of eggs ( Ratnieks and Keller 1998 ; Abe et al. 2009 ). Assessment of the primary sex ratios of the parasitoid *Melittobia austral-ica* showed that sex allocation is under precise control with the sexes produced in a regular sequence throughout the period of oviposition ( Abe et al. 2009 ). The microsatellites developed in the present study have also been directly applied to the molecular-genetic detection of haploid (male) and diploid (female) eggs in *G. legneri* ( Khidr et al. 2013 ). This provides an evaluation of primary sex ratios that is unbiased by developmental mortality (a longstanding obstacle in sex allocation research on many species, e.g., Fiala 1980; Hardy and Cook 1995 ; Hardy et al. 1998 ; Krackow and Neuhäuser 2008 ; Abe et al. 2009 ). The consistent between-strain polymorphisms (U and C-field) and cross-mated mothers were utilized, such that haploid and diploid eggs had different marker compositions. This work showed, for instance, that relationships between sex ratio and group size can be obscured by developmental mortality when the sex of eggs is not assessed directly, and also that male and female eggs may tend to be laid in spatial separation ( Khidr et al. 2013 ).

In conclusion, the *G. nephantidis* laboratory culture evaluated was found not to be polymorphic in terms of the 12 microsatellite markers presently developed. This likely reflects the limited genetic variability within this population but may be due to a prolonged period in laboratory culture. For *G. legneri* , no polymorphisms were found within strains using the 23 designed markers. As some strains were recently collected from the field, this finding suggests natural genetic variation is locally limited. However, there were six primers that showed clear between-strain marker polymorphism in *G. legneri* . Six markers differed between strains collected recently in Chile and strains believed to originate from Uruguay several decades ago, while the two Chilean strains differed in only one microsatellite marker.

These markers have already proved useful for experimental work on kin recognition mechanisms, as they show that females from different strains genuinely derive from populations with a different genetic background, and also for studies on sex allocation strategies, as consistent between strain polymorphisms allow the molecular-genetic detection of haploid (male) and diploid (female) eggs of cross-mated mothers.

## Abbreviations

SSR: simple sequence repeat
